# Supplementary material for: Bee Venom Melittin Protects against Cisplatin-Induced Acute Kidney Injury in Mice via the Regulation of M2 Macrophage Activation
Source: Toxins (Basel). 2020 Sep 6;12(9):574. doi: 10.3390/toxins12090574 (PMC7551791; doi:10.3390/toxins12090574)
Supplement: Supplementary file 1 [file toxins-12-00574-s001.pdf]

Supplementary Materials: Bee Venom Melittin Protects Against Cisplatin-Induced Acute Kidney Injury in Mice via the Regulation of M2 Macrophage Activation

Hyunseong Kim, Jin Young Hong, Wan-Jin Jeon, Seung Ho Baek and In-Hyuk Ha

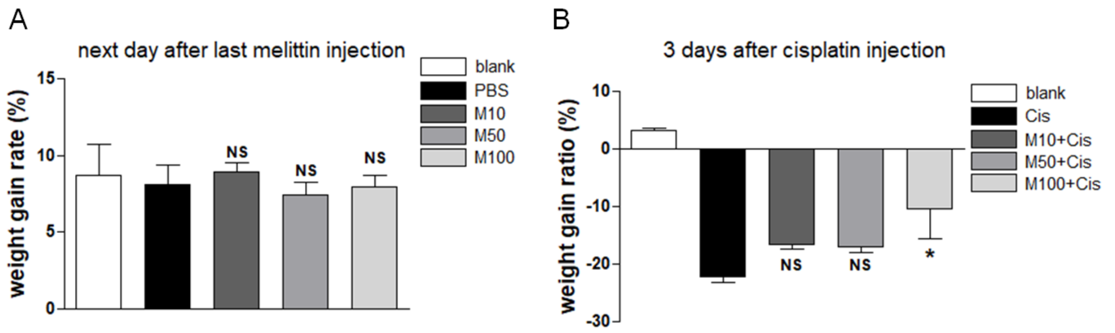

Figure S1. Weight gain rate of mice after melittin or cisplatin injection. (**A**) Percentage of the weight gain rate in the melittin groups with various concentrations compared to the control group (PBS-injected mice) without cisplatin administration after daily injection for 5 days ($n = 8$). (**B**) Percentage of the weight gain rate (%) in the melittin groups with various concentrations compared to the control group (PBS-injected mice) at 3 days after cisplatin administration. Data are expressed as the means ± standard error of the mean (SEM). Significant differences indicated as *$p < 0.05$ vs the control group were analyzed via one-way analysis of variance post-hoc test. (ANOVA) with Tukey's.

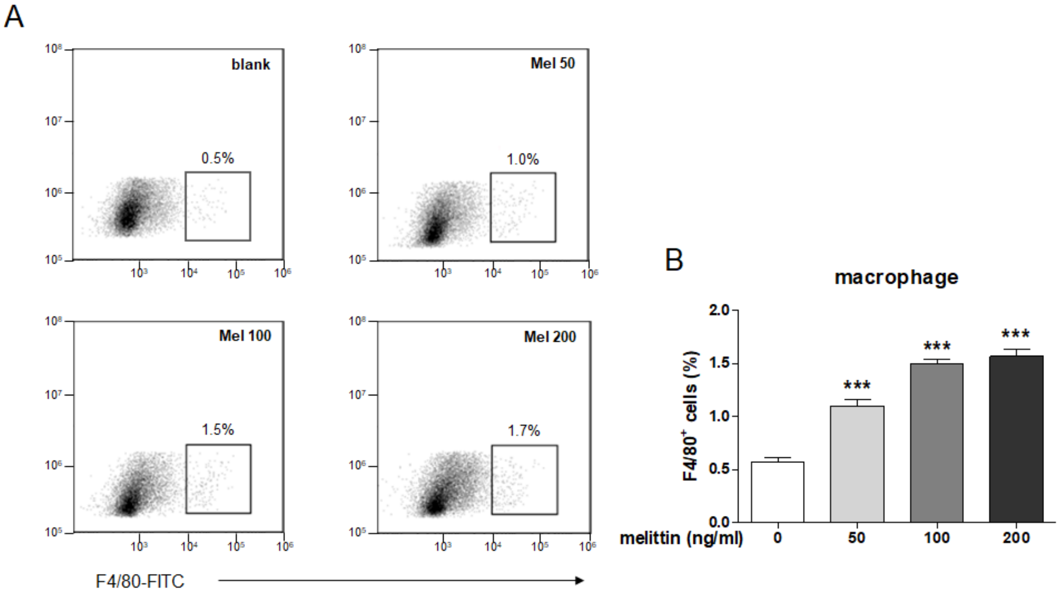

Figure S2. Effects of melittin on macrophage populations in mouse splenocytes. (**A**) Representative flow cytometry gating shows macrophages (F4/80) population in mouse splenocytes at 2 days after treatment of melittin with various concentration (50, 100, or 200 ng/ml). Quantification of the percentage of cells positive for (**B**) F4/80 macrophages relative to that in the blank group ($n = 6$). Significant differences indicated at ***$p < 0.001$ vs the blank group were analyzed via one-way ANOVA with Tukey's post-hoc test.

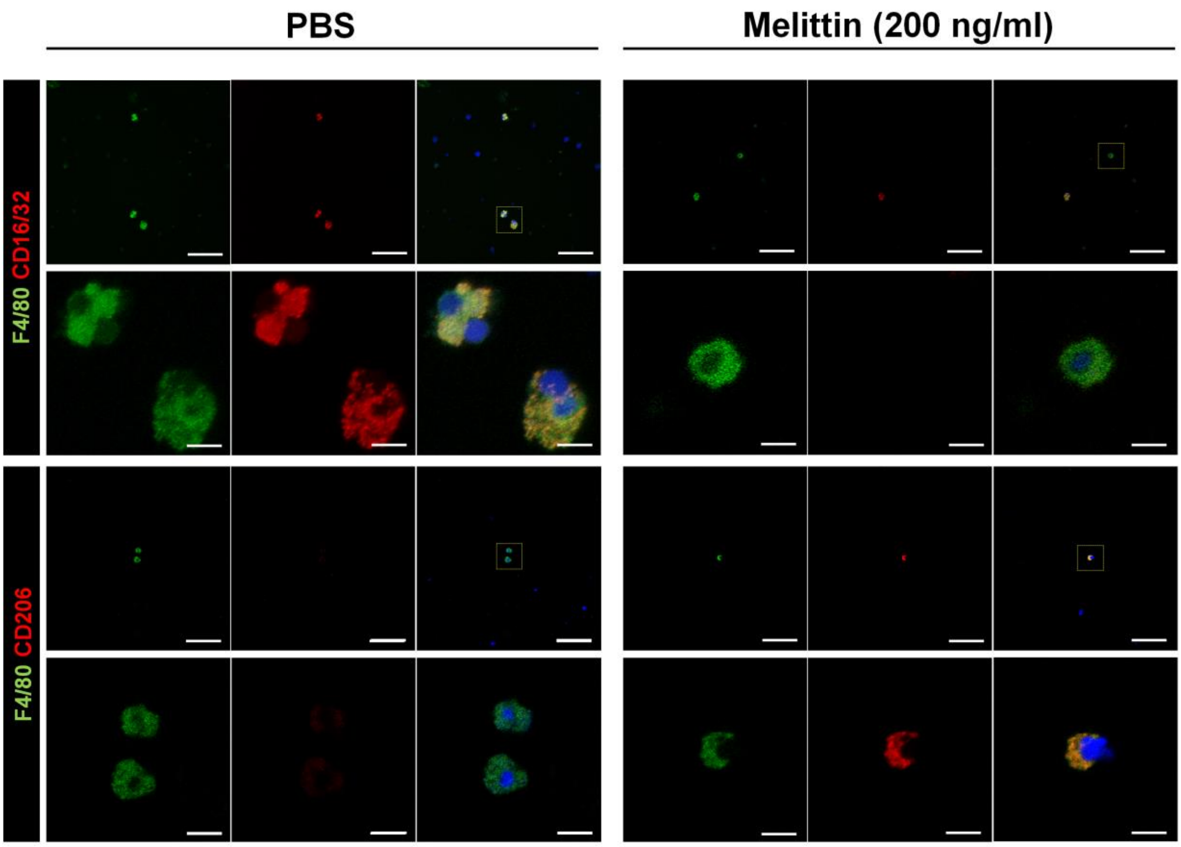

Figure S3. Representative images of CD16/32 (M1 macrophage) or CD206 (M2 macrophage) double stained with F4/80 macrophages marker within the splenocytes in control and melittin group treated with 200 ng/ml.

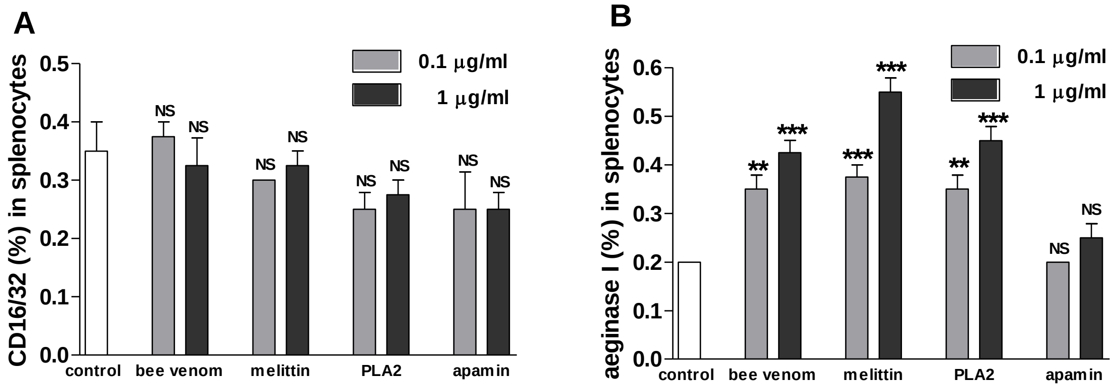

Figure S4. M1/M2 population change by bee venom and its constituents. Quantification of the percentage of cells positive for (**A**) CD16/32 (M1 macrophage marker) and (**B**) Arg1 (M2 macrophage marker) in splenocytes treated with the major constituents of bee venom (melittin, PLA2, apamin) relative to that in the blank group using flow cytometry ($n = 6$). Significant differences indicated at $^{**}p < 0.01$, and $^{***}p < 0.001$ vs the blank group were analyzed via one-way ANOVA with Tukey's post-hoc test.

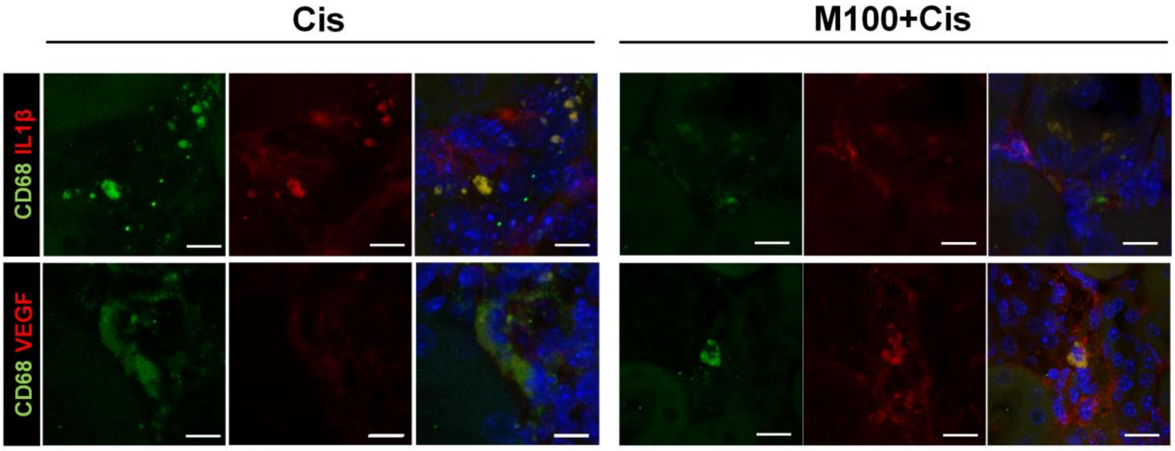

Figure S5. Representative images of IL-1β (M1 macrophage) or VEGF (M2 macrophage) double stained with CD68 macrophages marker within the renal tissue in cisplatin and melittin preinjected group with 100 μg/kg in presence of cisplatin. Scale bar = 50 μm.
